# Lethal Complications and Complex Genotypes in Shwachman Diamond Syndrome: Report of a Family with Recurrent Neonatal Deaths and a Case-Based Brief Review of the Literature

**DOI:** 10.3390/children11060705

**Published:** 2024-06-07

**Authors:** Danai Veltra, Nikolaos M. Marinakis, Ioannis Kotsios, Polyxeni Delaporta, Kyriaki Kekou, Konstantina Kosma, Joanne Traeger-Synodinos, Christalena Sofocleous

**Affiliations:** 1 Laboratory of Medical Genetics, School of Medicine, National and Kapodistrian University of Athens, “Agia Sophia” Children’s Hospital, 11527 Athens, Greece; dveltra@med.uoa.gr (D.V.); nikomari@med.uoa.gr (N.M.M.); kkekou@med.uoa.gr (K.K.); kokosma@med.uoa.gr (K.K.); csofokl@med.uoa.gr (C.S.); 2 Research University Institute for the Study of Genetic and Malignant Disease of Childhood, “Agia Sophia” Children’s Hospital, 11527 Athens, Greece; 3 Neonatal Intensive Care Unit, “Hippocration” General Hospital, 54642 Thessaloniki, Greece; 4 Thalassemia Unit, First Department of Pediatrics, National and Kapodistrian University of Athens, “Agia Sophia” Children’s Hospital, 11527 Athens, Greece; pdelaporta@med.uoa.gr

**Keywords:** neonatal deaths, SBDS, complex underlying genotype

## Abstract

Shwachman Diamond Syndrome (SDS) is a multi-system disease characterized by exocrine pancreatic insufficiency with malabsorption, infantile neutropenia and aplastic anemia. Life-threatening complications include progression to acute myeloid leukemia (AML) or myelodysplastic syndrome (MDS), critical deep-tissue infections and asphyxiating thoracic dystrophy. In most patients, SDS results from biallelic pathogenic variants in the *SBDS* gene, different combinations of which contribute to heterogenous clinical presentations. Null variants are not well tolerated, supporting the theory that the loss of SBDS expression is likely lethal in both mice and humans. A novel complex genotype (SBDS:c.[242C>G;258+2T>C];[460-1G>A]/WFS1:c.[2327A>T];[1371G>T]) was detected in a family with recurrent neonatal deaths. A female neonate died three hours after birth with hemolytic anemia, and a male neonate with severe anemia, thrombocytopenia and neutropenia succumbed on day 40 after *Staphylococcus epidermidis* infection. A subsequent review of the literature focused on fatal complications, complex SBDS genotypes and/or unusual clinical presentations and disclosed rare cases, of which some had unexpected combinations of genetic and clinical findings. The impact of pathogenic variants and associated phenotypes is discussed in the context of data sharing towards expanding scientific expert networks, consolidating knowledge and advancing an understanding of novel underlying genotypes and complex phenotypes, facilitating informed clinical decisions and disease management.

## 1. Introduction

Shwachman Diamond Syndrome (MIM#260400) was first described in 1964 by Shwachman et al. [1], followed by Bodian et al. [2], as a new entity in patients with cytopenia and pancreatic hypoplasia resembling cystic fibrosis. SDS, also categorized as an inherited bone marrow failure syndrome (IBMFS), is a multi-system disease characterized by exocrine pancreatic insufficiency with malabsorption (often presented as diarrhea), neutropenia in infancy, skeletal anomalies such as metaphyseal chondrodysplasia and aplastic anemia [3,4,5,6]. Heterogenous clinical presentations are often recorded to include additional characteristics such as intrauterine growth retardation (IUGR), neurocognitive dysfunction [7], neurodevelopmental impairments and intellectual disabilities of variable severity, and non-specific neuroimaging findings. Disruptions of other organs or systems involving teeth, liver, kidneys and the immune system may also become evident in SDS [3,4,8,9]. Immunologic manifestations may include inflammatory bowel disease [10], psoriasis, systemic lupus erythematosus, hemophagocytic lymphohistiocytosis autoimmune-like liver disease, duodenal inflammation [9,11,12,13,14,15] and other inflammatory conditions such as blepharoconjunctivitis, arthritis, chronic recurrent multifocal osteomyelitis and scleroderma [16]. High infection risk in SDS is possibly attributed to lower neutrophil counts and B-cell/T-cell defects, chemotaxis as well as to a proposed association of SDS with the hyperactivation of mTOR and STAT3 [17,18,19], and may explain the severe presentations of infectious diseases. Rat-bite fever, an important bacterial zoonosis primarily caused by Streptobacillus moniliformis, was reported in a female adolescent SDS patient requiring mechanical ventilation for 6 days and hemodialysis for 6 weeks while also presenting with purpuric lesions, sepsis and extended necrotic areas on the arm and forearm. Altered immune regulation from increased mTOR activity in SDS was proposed to contribute to the severe presentation of the patient [20]. Endocrine disorders in SDS include insulin-dependent diabetes, hypogonadotropic hypogonadism, hypothyroidism and growth hormone deficiency (reviewed in [21]). Growth parameters such as height and weight are usually restricted (below the third percentile) even in cases where pancreatic enzyme replacement is followed. Profound infantile hypotonia, manifesting as a primary symptom, has also been recorded and can complicate differential diagnosis [22,23,24]. 

The diagnosis of SDS is suspected with exocrine pancreatic dysfunction and bone marrow failure, and, in more than 90% of patients, is confirmed upon the detection of biallelic pathogenic variants in the *SBDS* gene (MIM#607444). Pathogenic or likely pathogenic variants, biallelic in *DNAJC21* (MIM#617048) and *EFL1* (MIM#617538) genes and monoallelic in *SRP54* (MIM#604857), have also been linked to SDS [25,26,27].

The *SBDS* gene spans ~7.9 kb and has five exons that encode a predicted protein of 250 amino acids. The gene is located within a segment of 305 kb on 7q11.21 and is located 5.8 Μb proximally to a duplicated and inverted copy, the SBDSP pseudogene. SBDSP, although presenting 97% homology with SBDS, has variations such as nucleotide changes or deletions that hinder the translation of the transcribed RNA to a full-length protein [28]. Following structural analysis studies, the SBDS protein was shown to comprise a conserved N-terminal FYSH domain with a potential nuclear localization signal and a specific nuclear export activity for FYSH, a central helix-turn-helix domain and a C-terminal domain homologous to an RNA-binding motif [29,30,31,32]. 

The SBDS protein appears to be involved in ribosome biogenesis and more specifically in cytoplasmic ribosome maturation [26,33,34], indicating that SDS may be categorized as a ribosomopathy. Abnormal synthesis or the loss of the SBDS protein was, over the years, proposed to have variable impacts, including defective spindle formation and the subsequent increased incidence of aneuploidy [35], Fas ligand-induced apoptosis [36], hypersensitive response to cellular stresses [37], impaired osteoclastogenesis disrupting bone homeostasis [38] and decreased MYC expression leading to progenitor proliferation failure [34]. SBDS-EFL1 (Elongation Factor Like-1), triggering the GTP-dependent release of elF6 (eukaryotic initiation factor 6) from 60 S pre-ribosomes in the cytoplasm, induce the translational competence of ribosomes, requiring 80 S ribosome assembly and elF6 recycling to the nucleus. SBDS serves as the nucleotide exchange factor for EFL1, thereby increasing its affinity to GTP over GDP [39,40].

The majority (75%) of patients are compound heterozygotes for two pathogenic variants, including SBDS:c.183_184delTAinsCT and SBDS:c.258+2T>C, both considered to be the result of gene conversion between SBDS and SBDSP [8]. Further loss-of-function alterations have been detected in all five exons of SBDS, some of which are recurring in apparently unrelated families. Missense *SBDS* variants may be classified in those affecting protein stability mimicking a rather typical loss-of-function mechanism and those allowing intact protein fold, although altering surface epitopes [26,40,41]. Of importance, distinct open and closed conformations of the protein are required for proper function, governed by an allosteric mechanism involving the N- and C-terminal domains [40].

Although there are no clear genotype–phenotype correlations associated with SDS [42], the homozygosity of the null SBDS:c.183_184delTAinsCT allele is already acknowledged as incompatible with life [8]. 

To contribute knowledge towards predicting the consequence of SDS variants, a novel complex SBDS genotype (SBDS:c.[242C>G;258+2T>C];[460-1G>A]) is herein described in a family with recurrent neonatal deaths. For further evaluation of complex SBDS genotypes and/or unusual clinical presentations, a review of the literature revealed rare/sporadic cases of lethal complications in neonates and infants, while interesting cases with unexpected combinations of genetic and clinical findings were also recorded. Acknowledging the presence of non-common genotypes and associated phenotypes highlights the need for data sharing, especially in view of detecting analogous cases in the future.

## 2. Case Presentation

**Figure 1:**
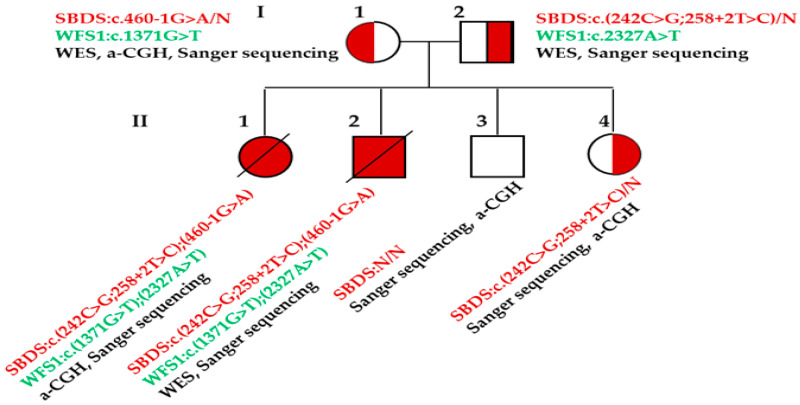
Family tree presenting the methodology used and genetic findings for each member. A novel and complex genotype, SBDS:c.[242C>G;258+2T>C];[460-1G>A], was revealed in probands II-1 and II-2. Prenatal diagnosis on naturally conceived subsequent pregnancies disclosed a normal fetus (II-3) and a carrier of the paternal variants (II-4), leading to the birth of a healthy male and a healthy female, respectively.

A male neonate (II-2, Figure 1), prenatally diagnosed with IUGR and born at 38 weeks of gestation via Caesarian section, was referred for targeted *SBDS* gene analysis due to severe anemia, thrombocytopenia and neutropenia (Hct: 0.196; Hb: 63 g/L; PLT: 11 × 10^9^/L; WBC: 9.2 × 10^9^/L; neutrophils: 0.5%), evident from the first day of life. Bone marrow aspiration was not performed , probably due to the severe presentation of the newborn . He was the second child of non-consanguineous Greek parents and presented additional respiratory distress and shock. During hospitalization, he tested positive for *Staphylococcus epidermidis* and died forty days after birth despite support with mechanical ventilation, daily transfusions and treatment with growth factor (G-CSF). From the family history, the first child (II-1, Figure 1), a female, was born prematurely due to IUGR at 32 weeks of gestation and succumbed within the first three hours of life due to pancytopenia and hemolytic anemia. Prenatal array comparative genomic hybridization (a-CGH) analysis (SurePrint G3 CGH v2 microarray Kit 8 × 60-Agilent (Agilent, Santa Clara, CA, United States), performed in another laboratory), instigated after the detection of short limbs (femur and humerus) and fetal echogenic bowel, did not reveal any pathogenic or likely pathogenic copy number variations (CNVs).

Postmortem laboratory workup and autopsy revealed paleness, petechiae and a hydropic placenta. Table 1 summarizes the clinical findings recorded in both neonates. 

Parental Whole Exome Sequencing (WES) (performed in Dante Labs, New York, NY, USA, with an Illumina sequencer with the BGI exome kitV459M (BGI, Yantian District, Shenzhen, China)) reported heterozygosity for the SBDS:c.258+2T>C pathogenic variant in the paternal sample and no pathogenic variants in the mother; CGH on the maternal sample had apparently excluded pathogenic CNVs. Bioinformatic re-analysis of the WES (.vcf) files in a second private genetics laboratory in Greece confirmed the previous variant on the paternal sample, and additionally detected the heterozygous pathogenic variant SBDS:c.460-1G>A in the maternal sample. Of interest, SBDS:c.460-1G>A, a pathogenic variant first recorded in 2005 [31], was not listed in the ClinVar database before 2020. The variant presents with a relatively high prevalence (5 of 12 cases, 41.66%) in the Greek population, [9] but, to the best of our knowledge, it was never detected in homozygosity or compound heterozygosity with SBDS:c.183_184delTAinsCT [8,9,10]. Upon the referral of the second child (II-2) to the Laboratory of Medical Genetics, University of Athens, parental samples and DNA from proband II-1 were requested along with the previous (.vcf) files. 

DNA was extracted from peripheral blood lymphocytes, buccal swab and hair roots of the male proband (day 1 of life) and from hepatic, spleen, vertebra and bone marrow postmortem biopsies (day 40). For the female neonate, a DNA sample was provided from the laboratory performing the prenatal diagnosis on chorionic villus sampling. Direct Sanger sequencing of the *SBDS* gene, including all exons and flanking sequences, was performed and analyzed on an ABI Prism 3500 Genetic Analyzer (Applied Biosystem, Woburn, MA, USA). 

Based on the clinical manifestations of both neonates, generally far severer than that expected for SDS, further investigation included the following:

a. Bioinformatic re-analysis of parental vcf files in search of variants related to phenotypes, including hematological defects, bleeding disorders, anemias, aplastic anemias or neonatal death, as well as variants involving genes associated with autosomal recessive disorders, where both parents were to be found carriers.

b. WES on the DNA extracted from hepatic tissue biopsy from the deceased male neonate (II-2) used the Human Core Exome kit (Twist Bioscience^®^, San Francisco, CA, USA). The resulting libraries were subjected to paired-end sequencing on an Illumina NextSeq^®^ 500 (Illumina, San Diego, CA, USA). Library preparation and sequencing were outsourced (Genotypos-Science Labs, Athens, Greece). ES data quality acceptance parameters included a mean depth of coverage >50× with >98% regions at 25×. Variant annotation was performed with the ANNOVAR algorithm and variant filtration with the VarAFT v2.16 Variant Analysis and Filtration Tool (http://varaft.eu, last assessed on 14 May 2024). Variant classification followed the recommendations of the American College of Medical Genetics (ACMG) guidelines [43].

**Figure 2:**
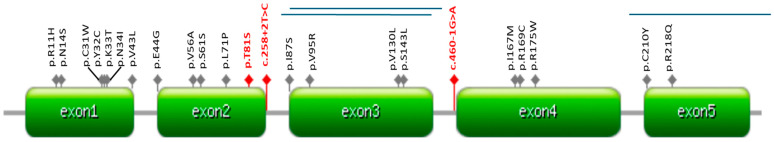
Prosite graphic presentation of the *SBDS* gene and some of the missense variants recorded in cases with typical or atypical presentations of Shwachman Diamond Syndrome. Selected variants include those reported in ClinVar and LOVD as pathogenic or likely pathogenic and are in black; variants in red are the ones detected in the present family; blue lines represent large deletions described in the literature [45,46,47].

Direct sequencing of the *SBDS* gene revealed the same genotype for both siblings, disclosing three variants, including the previously identified SBDS:c.258+2T>C and SBDS:c.460-1G>A variants, and an additional novel variant, the SBDS:c.242C>G (Figure 2). Analysis of the parental samples indicated that variants SBDS:c.258+2T>C and SBDS:c.242C>G were in cis and of paternal origin, while SBDS:c.460-1G>A was maternal. To the best of our knowledge, the SBDS:c.242C>G:p.Thr81Ser missense variant has never been reported in the literature and could only be ascertained in silico with prediction tools. According to the ACMG guidelines [43], it was characterized as a variant of unknown significance (VUS, PM2, PP2). Bioinformatic re-analysis indicated that both parents were also carriers of distinct variants in the *WFS1* gene, known to be related to the rare autosomal recessive Wolfram syndrome type 1 (MIM#606201). Variants WFS1:c.1371G>T (maternal) and WFS1:c.2327A>T (paternal), both located in the “hot spot” exon 8, were previously described in unrelated families [44]. Targeted Sanger sequencing of *WFS1* confirmed the findings on parental samples and disclosed compound heterozygosity in both siblings.

WES analysis performed on the DNA extracted from the hepatic cells of postmortem liver biopsy from the deceased male infant (II-2) revealed no other pathogenic or likely pathogenic variants for anemias, aplastic anemias, thrombocytopenia, neutropenia, neonatal breathing dysregulation, IUGR, abnormalities of the ulna and femur, cardiomyopathy and neonatal death (Table 2). Besides the use of these human phenotype ontology (HPO) terms, bioinformatic analysis focused specifically on the genes implicated in similar presentations, including *DNAJC21*, *EFL1*, *SRP54*, *EIF6* and *TP53*, where no variants were revealed.

## 3. Discussion

SDS is an autosomal recessive genetic disease, although a higher incidence and a more severe phenotype is recorded in male patients [5]. Significant clinical heterogeneity is recorded among patients with the same genotype and even between affected siblings. Direct genotype–phenotype correlations may not be feasible in SDS [48], but the advent of next-generation sequencing approaches may help recognize genetic disease modifiers as well as the possible implication of additional environmental factors. Of interest, such tests have already indicated an ameliorating effect of somatic (or even germline) variants which abolish the expression of an EIF6 allele, whereby SDS patients present with improved hematological findings and reduced risks of malignancies [49,50]. Conversely, poor prognosis with severe outcomes and a short survival rate in patients with biallelic *SBDS* variants were recorded in the presence of somatic *TP53* variants [51].

Extreme clinical presentations, not usually observed in SDS syndrome, were recorded in the family described herein. Given the co-inheritance of variants linked to Wolfram syndrome (WS), it could be questioned whether the aggravated phenotype was the result of the co-existing syndromes. WS is a rare neurodegenerative disorder characterized by diabetes, optic atrophy, and deafness, with a later onset (mean age at 6 years) and an average age of death at around 30 years [52,53]. WFS1:c.2327A>T was first described in a teenage girl with diabetes mellitus, optic atrophy, cochlear implant, depression and apnea episodes [54], and WFS1:c.1371G>T in 40-year-old twin males, one of whom died after stroke [55]. The gradually increasing blood glucose levels recorded in the male neonate (2.55 mmol/L on day 1 to 9.98 mmol/L on day 30) is a finding pointing to WS; however, previous reports hypothesized that altered immune regulation in SDS may impact the manifestation of type 1 diabetes mellitus [56,57,58,59]. Impaired insulin secretion predisposing SDS patients to dysglycemia is also recorded and attributed to defective ribosome function [60]. Finally, postmortem detection of cardiomyopathy could be attributed to either WS or SDS, further complicating the final resolution. Whether a combination of *SBDS* and *WFS1* genotypes could explain the observed phenotype remains elusive.

SBDS, the protein deficient in SDS, plays an important role in the maturation of the 60 S ribosomal subunit. In studies on mouse models where Sbds^+/−^ mice were cross-bred, no homozygous (Sbds^−/−^) offspring were recorded, suggesting that Sbds is essential for embryonic viability [61]. When pathogenic variants allow for the preservation of some SBDS activity, an SDS phenotype develops, while in the case of homozygous null alleles like SBDS:c.183_184delTAinsCT:p.Lys62X, no SBDS activity is present and embryonic death occurs [62]. SBDS:c.460-1G>A is a splice variant which, when combined to SBDS:c.258+2T>C, affects protein synthesis, as shown by Western blots of lymphocytes lacking the full-length SBDS protein [63]. The SBDS:c.258+2T>C common variant is predicted to give rise to a cryptic splice site at positions c.251_252, resulting in a truncated and non-functional protein, p.(C84fs*3) [8]. However, studies have shown that, in ~10% of the transcribed RNA, normal splicing occurs and retains some of the functional SBDS protein, which may explain the high survival rates of c.258+2T>C homozygous embryos [62]. More recent findings, showing that exon 2 is rich in splicing regulatory elements and cryptic splice sites, confirmed aberrant splicing mechanisms, whereby transcripts lacking exon 2 were traced in control cells from normal subjects, and correct transcripts (~2%), allowing residual SBDS expression, were traced in cells with the variant [64]. SBDS:c.242C>G, a novel variant of unknown significance, is located in exon 2 and causes a threonine-to-serine substitution at residue 81 p.(Thr81Ser) located within the N-terminal domain (FYSH) of the protein. This domain contains a mixed α/β fold implicated in the RNA metabolism and is considered the most frequently mutated region of the protein. Missense variants (Figure 2) affecting the residues sited within the hydrophobic core of FYSH include the already known disease-causing SBDS:p.Cys31Trp, SBDS:p.Leu71Pro and SBDS:p.Ile87Ser variants, shown to disrupt protein stability and function [31]. In silico analysis of the SBDS:c.242C>G novel variant using the VarMap software tool indicates possibly damaging propensities [65]. Although both threonine and serine share similar structures, including neutral acid chains, they seem to have inherent structural differences when subjected to modifications like phosphorylation and glycosylation. These can have an impact on both the local protein backbone, the protein–protein interactions and the surrounding water shell, and indicate that a somewhat deleterious effect may be predicted for the SBDS:p.Thr81Ser variant [66,67]. With respect to the acknowledged modifying nature of the *EIF6* and *TP53* variants, no variants in these genes were recorded in the DNA sample extracted from the postmortem liver biopsy of proband II-2 undergoing WES. Functional studies are required to allow for the robust interpretation of variant pathogenicity, but are not currently feasible since the samples available from the affected siblings do not meet the requirements of mRNA and protein studies.

In an effort to better understand the consequences of the molecular data towards informed clinical decisions, a mini review of the literature was conducted in the context of complex or novel *SBDS* genotypes and their clinical impact, as well as of confounding conditions and differential diagnosis and life-threatening complications in SDS.

### 3.1. Rare Genotypes in SBDS

Complex genotypes involving the presence of more than two (likely) pathogenic variants were scarce. Uncommon molecular findings mostly concerned deletions of large regions in trans with one of the known *SBDS* variants. A large (19 kb) deletion removing part of intron 4, exon 5 and the 3′UTR of *SBDS* was reported in a patient from the National Cancer Institute: Inherited Bone Marrow Failure Syndrome Studies [45]. Compound heterozygosity of c.258+2T>C with c.258+374_459+250del (p.Ile87_Gln153del) was detected in a patient presenting with severe anemia, cyclic neutropenia, pancreatic exocrine insufficiency and skeletal abnormalities [46]. SBDS: c.[258+533_459+403del];[258+2T>C], a deletion encompassing exon 3, is a recurrent deletion reported in patients with variable clinical presentations of SDS [47]. Of interest, the variant was characterized as a “founder”, one originating independently twice in two different geographical places in Italy (Sicily and Lazio) as a result of gene conversion and non-homologous allelic recombination between *SBDS* and its pseudogene, SBDSP [68]. Thus, deletions involving *SBDS* should be investigated, especially when only one causative single nucleotide variant is detected in patients with SDS presentation.

Finally, the heterozygosity of rare or common *SBDS* variants has also been reported. NM_016038.3:c.127G>T:p.Val43Leu was detected to emerge as an autoimmune neutropenia mimicking a cardinal feature of SDS [69], and the common c.258+2T>C is thought to be associated to telomere shortening in leukocytes and acquired aplastic anemia [70].

### 3.2. Misleading Phenotypes/Confounding Entities

Atypical presentations and additional non-expected features have been recorded in SDS patients baring both novel and known variants, and complicate differential diagnosis.

Hepatic involvement is described in children with SDS, although it was reported as mild and resolves with age. However, severe cirrhosis was recorded in the following two cases: (a) one of a child initially presenting with failure to thrive and mildly elevated transaminase levels, attributed to pancreatic insufficiency due to SDS [71], and (b) an unusual case of extremely early neonatal cirrhosis in a 5-day-old male neonate admitted because of sepsis, hypoglycemia and feeding difficulties [72].

Endocrine manifestations in SDS include growth hormone deficiency and diabetes, but show such a wide variability that it possibly hinders the identification of a consistent endocrine SBDS phenotype [73]. A male newborn presenting with hypoglycemia and micropenis was also reported to have growth hormone and cortisol deficiencies. Subsequent diagnosis of congenital hypopituitarism was followed by hydrocortisone administration at 1 week of life and growth hormone at the age of 2 months. The patient presenting also with transient thrombocytopenia and subsequent intermittent neutropenia was eventually diagnosed with SDS due to the compound heterozygosity of SBDS:c.183_184delTAInsCT and c.258+2T>C [74].

Skeletal features such as a bell-shaped or long narrow thorax can be expected in a wide range of bony dysplasia syndromes. In the presence of accompanying respiratory distress, asphyxiating thoracic dystrophy (ATD, Jeune syndrome) could serve as a differential diagnosis. Skeletal anomalies in SDS mainly include metaphyseal chondroplasia and short stature, while findings such as clinodactyly, kyphosis, scoliosis, coxa vara, vertebral collapse, slipped femoral epiphysis, supernumerary metatarsals, genu and cubitus valgus, pes cavus and osteopenia have also been recorded [75]. Several reports describe patients who were initially diagnosed with ATD and were later shown to have SDS. In Keogh et al. [76], the patient, a female neonate, was misdiagnosed with ATD, probably because of the neonatal asphyxia recorded as the cause of death of her brother. Despite developing progressive pancytopenia, she was only diagnosed with SDS 11 years later upon detection of bone marrow failure, trilineage dysplasia, pancreatic insufficiency and heterozygous *SBDS* pathogenic variants [76].

Dermatologic findings such as eczema, variable pigmentation, dry skin [45], as well as a higher risk for acquired skin infections and/or abscesses [77] have been recorded in SDS. Skin eruptions were even recorded as the main symptom in a 6-month-old boy with ichthyosiform skin and eczema accompanied by failure to thrive [78]. In 2016, Scalais et al. reported on a female infant with early ichthyosis, associated dermal and epidermal intracellular lipid droplets, reminiscent of those described in Dorfman–Chanarin syndrome (DCS) or Wolman’s disease. Hypoglycemia was observed as early as 3 months, while neutropenia and pancreatic insufficiency were observed 11 months later. Genetic testing disclosed the presence of SBDS:c.258+2T>C in trans with the novel SBDS:c.284T>G:p.Val95Arg variant possibly leading to an impaired neutral lipid metabolism, most likely occurring in the cytoplasm compartment, analogous to DCS [79].

Neurodevelopmental impairments and intellectual disabilities of variable severity, as well as other psychological impairments, are becoming widely recognized in SDS [3,7,45,80,81]. Taha et al. reported on SDS siblings who, although having the exact same SBDS variants, c.[258+533_459+403del];[258+2T>C], presented different phenotypes. Besides the evident pancreatic insufficiency common in both siblings, other phenotypic features were notably heterogeneous. Markedly more severe, the male proband presented with enlarged metaphyseal regions and dysmyelopoiesis, which, however, neither he nor his sister required blood transfusion or supportive G-CSF therapy. Mild developmental delay with difficulties in expressive language and memory accompanied with minor facial dysmorphisms (hypertelorism and wide nasal bridge) and cryptorchism were also recorded, and were eventually attributed to a novel de novo *KMT2A* variant (c.10663G>A) [82]. Pathogenic or likely pathogenic *KMT2A* variants were associated with autosomal dominant Wiedemann–Steiner syndrome (MIM#605130), characterized by distinctive developmental delay, skeletal anomalies and short stature, that can also be recorded in SDS, as well as facial dysmorphism, horseshoe kidney and ocular, cardiac and dental manifestations [83].

### 3.3. Life-Threatening or Lethal Complications and Neonatal Deaths

Although many SDS patients are known to have extensive productive lives [3], early death has been reported to result from severe respiratory and hematologic complications [62]. Infantile death in SDS has also been related to malabsorption, severe bacterial infections and thoracic dystrophy, while, in older patients, mortality has mostly been associated with hematologic abnormalities [84].

Complications, including severe cytopenia of all blood cell lines, progression to AML or MDS as well as critical deep-tissue infections or asphyxiating thoracic dystrophy due to rib cage restriction, can become life threatening in patients with SDS as early as infancy [4,5,77,85,86]. Secondary to thoracic dystrophy, restrictive pulmonary disease, malabsorption and infections compromise longevity and life quality [21].

A French national cohort proposed that very early onset of cytopenia (even mild anemia or thrombocytopenia) relates to a high risk of severe malignant or non-malignant hematologic complications [87]. Severe cytopenia and bone marrow failure usually develop within the first decade of life. Clonal evolution to MDS/AML presents between the second and third decade of life, and the ~11% to 12% of SDS patients that will, at some point of their life, require hematopoietic stem cell transplantation (HSCT) may benefit from innovative approaches which can address poor response to transplantation [88]. Another, more recent, multi-institutional retrospective study on SDS patients with myelodysplastic syndrome or acute myeloid leukemia recorded a median survival of less than 12 months from the leukemia diagnosis and 7.7 years from that of myelodysplastic syndrome [89]. Finally, Furutani et al. concluded that hematologic complications presented the major cause of mortality (17/20 deaths; 85%) based on a study of 153 patients from the North American Shwachman Diamond and the Bone Marrow Failure Registries [90].

Cardiomyopathy, congenital heart disease and neonatal cardiac manifestations, like fibrosis revealed on histopathology, or depressed left ventricular contractility during exercise and subtle right ventricular diastolic dysfunction, have also been associated with SDS [91,92]. Myocardial necrosis seen on histopathology was related to an increased risk of infantile cardiac failure and death [93]. Frequent viral(and other) infections of infancy may lead to myocardial complication, such as viral myocarditis, potentially becoming life threatening. In the case of no inflammatory signs, fatal cardiomyopathy may also be linked to SDS, indicating the need for cardiac monitoring in both younger and older patients [94].

As already acknowledged, neonatal or early infantile death is uncommon. A few studies report death as a result of respiratory distress either closely after birth or within the first year of life [76,95], or Lethal Sedaghatian-type spondylometaphysial dysplasia [86] or cases with confounding conditions such as co-inherited cystic fibrosis [96]. A 29-week preterm neonate who developed multi-organ failure resulting in ventilator-dependent respiration, transfusion-dependent progressive bone marrow failure, neutropenia, recurrent sepsis and total parenteral nutrition-dependent gastrointestinal failure finally succumbed at day 54. He was characterized as homozygous for SBDS:c.183_184delTAinsCT, a null variant which is commonly considered incompatible with life [97]. The neonate with extremely early neonatal cirrhosis, described by Reddy et al., also succumbed with fulminant liver failure and hepatic encephalopathy [72]. In older infants (7–15 months) with SDS, cardiac manifestations, including myocardial necrosis or fibrosis (specific for the left ventricle), were considered the cause of death [93].

### 3.4. Disease Management

No gene-based therapy, drugs or chemical compounds are yet available to treat SDS. Pancreatic enzyme replacement and fat-soluble vitamin supplementation can help with exocrine pancreatic insufficiency, whereby complications are minimized and about 40–60% of patients become pancreatic-sufficient with advancing age. Blood and/or platelet transfusions are proposed for managing anemia and thrombocytopenia. Hematologic transformation to MDS or AML and skeletal abnormalities leading to thoracic dystrophy call for procedures such as HSTC or surgical intervention, respectively. Recent approaches include gene-based therapies such as the translational read-through-inducing drugs developed to target nonsense variants, including the SBDS:c.183_184delTAinsCT null variant. Ataluren (PTC124) and an additional NV848 analogue were shown to restore full-length SBDS protein synthesis [98]. In addition, counteracting the other common c.258+2T>C variant by RNA therapeutics and Base/Prime Editing was currently proposed as the basis of an “SDS personalized therapy” using edited hematopoietic stem cells [64].

Carrier screening and informed reproductive choices are of great importance for the management of the disease [5]. Prenatal or preimplantation genetic tests may be performed in SDS families with a previously affected child. As an alternative, expanded carrier screening potentially allows for the detection of pathogenic variants related to autosomal recessive diseases, although there are challenging limitations, including the variability of methodologies for DNA analysis, pipelines for variant classification and strategies for variant reporting. For example, reporting variants only if they have been previously reported in selected databases could lead to missing novel or unreported pathogenic variants and, as with the family in this report, could compromise the accurate estimation of disease risk.

To this end, and to the best of our knowledge, no other cases of neonatal deaths and/or complex genotypes such as the one presented are hitherto reported. In the absence of additional pathogenic or likely pathogenic variants to explain the severe fatal presentation, this novel SBDS:c.[242C>G;258+2T>C];[460-1G>A] genotype is considered causative in both probands of the family. This report aimed to highlight the need for multidisciplinary research and the management of rare diseases, as well as the value of data sharing and the expansion of scientific expert networks to consolidate knowledge and advance the understanding of novel underlying genotypes and complex phenotypes.

## Figures and Tables

**Table 1:** Clinical presentations of neonates.

	Sex	IUGR	Prenatal u/s Findings	Gestation	Lifespan	Neutropenia/ Thrombopenia/ Pancytopenia	Anemia	Infections	Postmortem Findings
II-1	F	+	Echogenic bowel, short femur, short humerus	32^+6^ weeks	3.5 h	Reported severe (no counts available)	Yes (hemolytic)	Unknown	Hepatic hemosiderosis grade III and hydropic placenta compatible with hemolytic anemia
II-2	M	+	Echogenic bowel, short femur, short humerus	38 weeks	40 days	Severe neutropenia (neutrophils < 500)	Yes (Hb 6.3 g/dL)	*Staphylococcus epidermidis*	Hemorrhagic infiltrates, vessel congestion in all organs tested (brain, bone, tongue, myocardium, lungs, bowel, stomach, liver, spleen, pancreas and kidneys) and myocardium ischemic lesions compatible with cardiomyopathy

**Table 2:** HPO terms implemented in the bioinformatic analysis of the data from the Whole Exome Sequencing (proband II-2).

Anemia *	HP:0001903
Thrombocytopenia *	HP:0001873
Neutropenia *	HP:0001875
Cardiomyopathy	HP:0001638
Hypoplasia of the extremities	HP:0009815
Respiratory distress *	HP:0002098
Neonatal death	HP:0003811
Intrauterine growth retardation	HP:0001511
Pallor	HP:0000980
Pancytopenia *	HP:0001876
Petechiae	HP:0000967
Hemolytic anemia	HP:0001878
Hydropic placenta	HP:0011414
Bone marrow hypocellularity	HP:0005528
Hyperglycemia	HP:0003074
Aplastic anemia	HP:0001915
Hypomagnesemia	HP:0002917
Abnormal circulating calcium concentration	HP:0004363

* Denotes phenotypic features usually serving as criteria for the differential diagnosis of SDS.

## Data Availability

The data presented in this study are available on request from the corresponding author. The data are not publicly available due to restrictions.
